# Diversity and distribution of Chirostyloidea and Galatheoidea (Decapoda, Anomura) in the Southern Gulf of Mexico

**DOI:** 10.3897/zookeys.612.9492

**Published:** 2016-08-23

**Authors:** Ana Rosa Vázquez-Bader, Adolfo Gracia

**Affiliations:** 1 Laboratorio de Ecología Pesquera de Crustáceos, Instituto de Ciencias del Mar y Limnología, UNAM, Av. Universidad # 3000, Universidad Nacional Autónoma de México, CU, Ciudad de México, 04510, México

**Keywords:** Chirostilydae, Galatheidae, Munididae, Munidopsidae, Gulf of Mexico, depth, distribution, abundance, new records

## Abstract

We examined the diversity, abundance, distribution, and average size of squat lobsters collected during eight cruises conducted on the continental shelf and slope of the Gulf of Mexico (Mexican/USA border to the Caribbean Sea). Six species belonging to two genera of Chirostyloidea, and 25 species of four genera of Galatheoidea are reported. A total of 1513 specimens were obtained of which 95 were Chirostylidae, two Galatheidae, 285 Munidopsidae, and 1131 Munididae. Of the species collected, 13.8% were only known from Caribbean Sea. Three species of Chirostylidae—*Gastroptychus salvadori*, *Uroptychus capillatus*, and *Uroptychus spiniger*—as well two of Munidopsidae, *Munidopsis bradleyi* and *Munidopsis riveroi*, are recorded for the first time in the Gulf of Mexico. The upper bathymetric range of one species and the lower one for eight species are extended. Biological and ecological traits of squat lobsters in the southern Gulf of Mexico are also provided.

## Introduction

Squat lobsters (Chirostyloidea and Galatheoidea) are abundant, speciose, and distributed worldwide (Fierro et al. 2008; Kilgoure and Shirley 2014; Konishi and Saito 2000; de Melo-Filho 2006; de Melo-Filho and de Melo 1992; de Melo-Filho and de Melo 2001 a and b; Miyake and Baba 1970; Navas et al. 2003; Pequegnat and Pequegnat 1970). Schnabel et al. (2011a) and Schnabel et al. (2011b) recognized ca.1000 species (ca. 10% undescribed) that occur in all marine habitats, from the intertidal zone to more than 5400 m depth, including anchialine caves and hydrothermal vents. Recent molecular and phylogenetic studies of the Anomura have proposed significant changes in the phylogenetic relationships of squat lobsters. These recent studies indicate that the Chirostyloidea Ortmann, 1892, include the following families: Chirostylidae Ortmann, 1892, Eumunididae A. Milne-Edwards & Bouvier, 1900, and Kiwaidae Macpherson, Jones & Segonzac, 2005 (Schnabel and Ahyong 2010). The Galatheoidea Samouelle, 1819, comprises: Galatheidae Samouelle, 1819; Munididae, Ahyong, Baba, MacPherson & Poore 2010; Munidopsidae, Ortmann, 1898; and Porcellanidae Haworth, 1825 (Ahyong et al. 2010; Schnabel et al. 2011a). The Chirostylidae currently includes seven genera and over 200 species worldwide (Baba 2009; Baba and Lin 2008; Baba et al. 2008; Ahyong et al. 2009, Schnabel and Ahyong 2010), whereas the Galatheidae includes 11 genera and 95 species; the Munididae 20 genera and 395 species; and the Munidopsidae four genera and 250 species (Ahyong et al. 2011).

Squat lobsters are of ecological and economic interest because they play an important role in the marine food chain in coastal areas and sustain important fisheries in Central and South America and the Mediterranean Sea. However, overall studies of squat lobsters around the world have been mainly related to new species descriptions, and relatively few studies have made emphasis on their ecology and population structure (Creasy et al. 2000; Huguet et al. 2005; Kassuga et al. 2008; Maiorano et al. 2013).

In the southern Gulf of Mexico, Chirostyloidea and Galatheoidea species composition, distribution and abundance are poorly known compared to the northern Gulf. Though they are one of the most abundant and diverse groups just after penaeoids, information is restricted to a few species records (Escobar et al. 2008). In the last decade, several expeditions to study benthic biodiversity of the continental shelf and slope of the southern Gulf of Mexico have been conducted (Gracia et al. 2010; Vázquez-Bader and Gracia 2013; Vázquez-Bader et al. 2014). This paper represents the first attempt to contribute to the overall diversity, ecology, and knowledge of this group in this area. We provide data about specific composition, depth and geographic distribution, as well as basic biology and ecology traits of squat lobsters in the southern Gulf of Mexico.

## Material and methods

**Figure 1.**
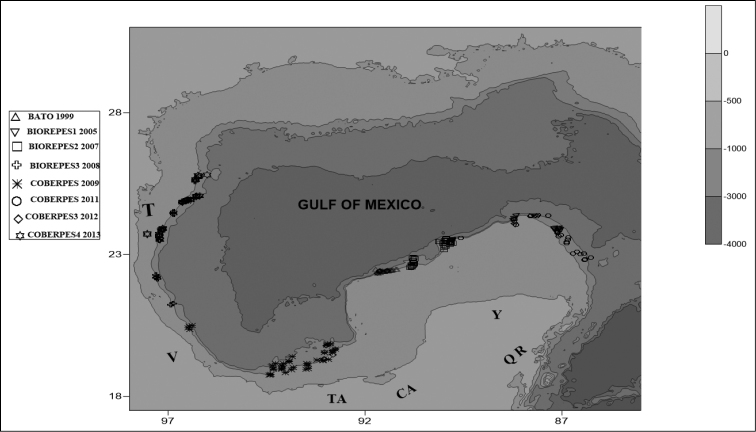
Study area showing sampling stations by oceanographic cruise. Abbreviations: T = Tampico, V = Veracruz, TA = Tabasco, C = Campeche, Y = Yucatán, QR = Quintana Roo.

All the material analyzed was collected during research cruises in the southern Gulf of Mexico onboard the R/V *Justo Sierra* of the Universidad Nacional Autónoma de México, on the continental shelf and slope of the Mexican Gulf of Mexico. Samples were obtained day and night with an otter trawl (18 m mouth aperture, 4.5 cm stretched mesh, 1.5 cm stretched mesh cod-end). Each tow lasted 30 min at a speed of 2.5–3.0 knots. We performed 273 trawls between 300 and 1200 m during the following expeditions: BATO (spring 1998), BIOREPES1 (summer 2005), BIOREPES2 (spring 2007), BIOREPES3 (autumn 2008), COBERPES (summer 2009), and COBERPES 2011 (spring 2011), COBERPES 3 (Autumn 2011), and COBERPES 4 (Summer 2012). Additional material examined was collected from cruises SGM (between 15–100 m) in the Campeche Bank area (Fig. 1).

The catch of each haul was sorted by species and counted onboard. All material collected was determined to species level, preserved in ethanol (80%), and deposited in the Crustacean Reference Collection of the Laboratorio de Ecología Pesquera de Crustáceos, Instituto de Ciencias del Mar y Limnología, UNAM
(EPC). For each specimen, we recorded cruise name and station number (stn.), and registered the geographic distribution according to a sector and subsector division within the Southern Gulf of Mexico (NNE = North northeastern; WNW = West Northwestern; SSW = South Sothtwestern; SSE = South Southestern; and ESE = East Southeastern) (Felder et al. 2009). We analyzed abundance, size and sex for each species (males = M and females = F). The carapace length (CL) was measured from the posterior border of the orbit to the mid-point of the posterodistal margin to the nearest 0.1 mm with a calibrated caliper. Differences in abundance by size and sex related to seasons (spring, summer and autumn) and 100 m depth intervals were analyzed for the most abundant species using a one-way ANOVA (STATISTICA version 12.0 StatSoft, Inc.). When ANOVA results were significant, post-hoc Tukey tests were used (Sokal and Rohlf 1995). Also, when sex ratio deviated from the expected proportion (1 M: 1 F), we applied a two tail χ^2^ test.

## Results

### Superfamily Chirostyloidea

We collected 95 individuals belonging to two genera and six species of Chirostylidae. Although all species occur throughout the Gulf of Mexico, only one had sample size large enough for a meaningful statistical analysis. Among the chryrostiloids examined we found approximately 31 individuals belonging to *Gastroptychus* that could not be clearly assigned any of described species of this genus. These specimens will be the object of a later study and are not considered herein in the total number for the family.

#### Gastroptychus salvadori

Taxon classificationAnimaliaDecapodaChirostylidae

Rice & Miller, 1991

##### Material examined.

BIOREPES 2 stn.12, 1 ovigerous female.

##### Remarks.

One ovigerous female was collected in June (CL 18.2 mm). This species was been reported for the Caribbean only by Baba et al. (2008). Thus, this is the first record for the Gulf of Mexico (SSW sector; off Alacranes Reef, Yucatán Peninsula). Also the upper bathymetric range is extended from 874 m to 650 m.

#### Gastroptychus spinifer

Taxon classificationAnimaliaDecapodaChirostylidae

(A. Milne-Edwards, 1880)

##### Material examined.

BATO stn. 20, 3 males; stn. 32, 1 male; stn. 33, 1 ovigerous female; stn. 34, 2 ovigerous females.

##### Remarks.

We collected only seven specimens (males CL 13.5–16.2; ovigerous females CL = 21.7–23.5 mm); 299–562.5 m. The ovigerous females distributed deeper (414.5–562.5 m) than males (299.0–311.8 m). These records are the first in the in SSW sector (Bank of Campeche), previous distribution was reported in NNE, NNW, and ESE of the Gulf of Mexico (Felder et al. 2009).

#### Uroptychus capillatus

Taxon classificationAnimaliaDecapodaChirostylidae

Benedict, 1902

##### Material examined.

COBERPES 2011 stn. B1, 3 females, 1 ovigerous female; stn. B9, 6 males, 8 females, 4 ovigerous females.

##### Remarks.

The 22 individuals occurred in front of Ría Lagartos, Quintana Roo between 976.0 and 1040.0 m. The overall sex ratio (1 M: 2.66 F) deviates significantly from the expected ratio 1:1 (χ^2^ = 4.545 with 1 degree of freedom, two-tailed (P = 0.03). The carapace length size in males ranged from 4.7 to 10.2 mm, whereas females range was 6.3–10.00 mm and in ovigerous females was 8.3 to 11.2. The material examined increased the lower bathymetric limit to 1040 m from previous range (306–573 m, Baba et al 2008). Also, it is the first record for the sector SSE Gulf of Mexico. Previous records are only for the Caribbean (Baba et al 2008).

#### Uroptychus nitidus

Taxon classificationAnimaliaDecapodaChirostylidae

(A. Milne-Edwards, 1880)

##### Material examined.

BATO stn. 29, 1 female; stn. 47, 1 female,1 ovigerous female; stn. 48, 1 female, 1 ovigerous female, stn. 53, 1 ovigerous female. BIOREPES 2 stn. 27, 1 male, 1 ovigerous female; stn. 28, 1 male; stn. 28b, 1 male, 1 ovigerous female; stn. 31, 1 female. BIOREPES 3 stn. A16, 1 ovigerous female; stn. C1, 6 males, 2 females, 3 ovigerous females. COBERPES stn. A6, 1 ovigerous female; stn. B10, 1 male; stn. B11, 1 male. COBERPES 2011 stn. B9, 15 males, 4 females, 5 ovigerous females, stn. D1b, 1 female, stn. D10, 1 female stn. C2, 1 male, 1 female, stn. D6b, 3 males, stn. C3, 3 males, 1 female.

##### Remarks.

This species was the most abundant and frequent of the genus *Uroptychus* (n = 62 individuals). Specimens were collected off Laguna Madre, Tamaulipas; Términos Lagoon, Campeche; Carmen y Machona, Tabasco; N of Alacranes Reef, and Progreso, Yucatán; between 352 and 1044 m. The maximum abundances were found in spring (58.12%) and in sector ESE (61.3%) between 406.5–1044.0 m depth. The overall sex ratio favored males 1.18 M: 1 F, but it was not statistically significant (χ^2^ = 0.581, with 1 degree of freedom, two-tailed P = 0.45). Ovigerous females were present in spring, summer, and autumn.

**Figure 2.**
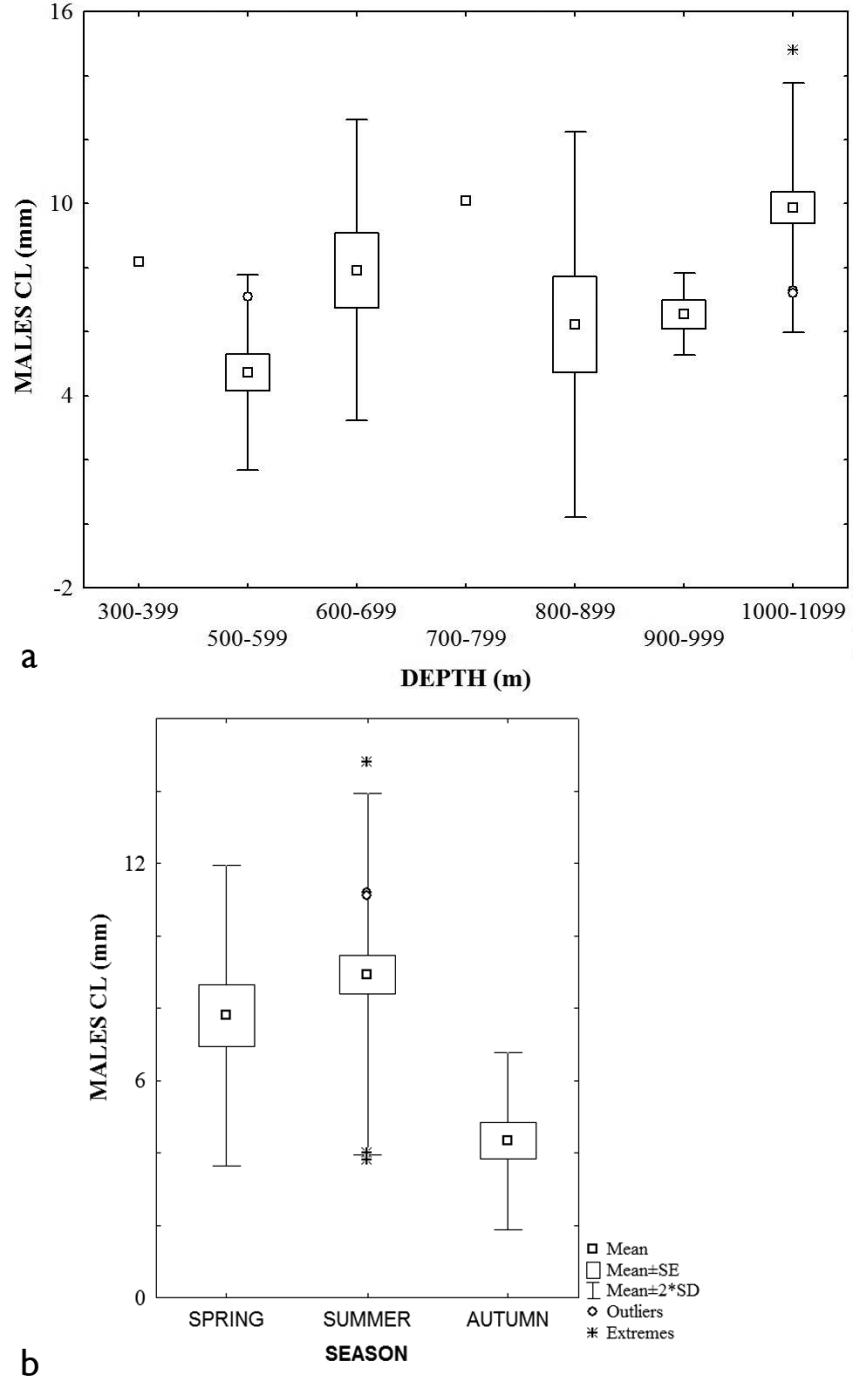
Size distribution of *Uroptychus nitidus*, males: **a** depth interval **b** season.

The bathymetric range was different in summer (352.0–1144.0) and autumn (510.0–552.0 m). Females presented a slightly larger mean carapace length x = 8.9 ± 3.45 (min. 5.0. max 12.9 mm) than males x = 8.8 ± 2.47 (min. 3.8. max 14.8 mm) and ovigerous females x = 8.4, ± 2.10 (min. 4.8 max 12.8 mm). However, ANOVA analysis were only significant for males, F _depth_ = 6.05, p = 0.00; F _season_ = 9.67, p = 0.00. The Tukey post hoc test, showed that summer was significantly different from autumn and spring. The largest mean size was found in summer whereas the smallest one was observed in autumn (Fig. 2b). Significant differences were found between 800–899 and 1000–1099 m depth interval and the others. Also, a size increasing trend related to depth was apparent (Fig. 2a).

#### Uroptychus spiniger

Taxon classificationAnimaliaDecapodaChirostylidae

Benedict, 1902

##### Material examined.

COBERPES 2011, stn. B9, 1 ovigerous female; COBERPES 4 stn. B10, 1 ovigerous female.

##### Remarks.

The only two ovigerous females (CL 10.3–11.9 mm) were reported off Laguna Madre, Tamaulipas; and off Isla Holbox, Quintana Roo, in a 990–1040 m depth range. These findings represent the first record in the Gulf of Mexico (WNW and ESE). Also the lower bathymetric limit was extended 332 m from the previous 708 m reported by Baba et al. (2008) in the Caribbean.

#### Uroptychus uncifer

Taxon classificationAnimaliaDecapodaChirostylidae

(A. Milne-Edwards, 1880)

##### Material examined.

COBERPES stn. A6, 1 ovigerous female.

##### Remarks.

We collected only one ovigerous female in summer at 1144 m. (CL 12.6 mm); that constitutes the first record in the sector SSW Gulf of Mexico (off Carmen y Machona Lagoon, Tabasco) This single record increases 654 m the reported bathymetric range (155 to 490 m, Baba et al. 2008; Felder et al. 2009).

### Superfamily Galatheoidea

We only collected two individuals of one genus and one species of Galatheidae family.

#### Galathea rostrata

Taxon classificationAnimaliaDecapodaGalatheidae

A. Milne-Edwards, 1880

##### Additional material examined.

SGM 10 stn. 145.78, 2 males.

##### Remarks.

*Galathea rostrata* was the only species collected of this family. The two individuals were found in front of Términos Lagoon, Campeche at 54 m depth (SSW). The two males (7.2–7.8 mm CL) were collected during autumn and constitute the first record in sector SSW.

#### Family Munididae

In this family we collected 1131 organisms belonging to two genera and nine species, of these, only five species had sample sizes large enough to stand statistical analyses.

##### Agononida longipes

Taxon classificationAnimaliaDecapodaMunididae

(A. Milne-Edwards, 1880)

###### Material examined.

BATO stn. 24, 1 male, stn. 26, 1 ovigerous female, stn. 27, 2 females, 5 ovigerous females, stn. 29, 3 males, stn. 33, 2 males, 3 females, 1 ovigerous female; stn. 41, 5 males, 1 female, stn. 49, 1 male; stn. 54, 1 female. BIOREPES 1 stn. 12, 2 males, 3 females, 1 ovigerous female; stn. 26, 5 males; stn. 27, 7 males, 3 females; stn. 30, 62 males, 61 females; stn. 31, 5 males, 1 female, 10 ovigerous females. BIOREPES 2 stn. 2, 2 males, 2 females, 1 ovigerous female; stn. 4, 1 ovigerous female; stn. 14, 1 male; stn. 15, 7 males; stn. 18, 1 female, 5 ovigerous females; stn. 34, 1 male, 1 female, 1 ovigerous females. BIOREPES 3 stn. A2, 3 males, 3 females; stn. A10, 1 female; stn. A22, 1 male; stn. A23, 1 ovigerous female; stn. A24, 2 males, 1 female; stn. A25, 1 male; stn. B2, 1 male, 2 females, 1 ovigerous female; stn. B3, 1 male, 2 females; stn. B6, 1 male, 1 female, 1 ovigerous female 1; stn. B7, 5 males, 2 ovigerous females; stn. C2, 1 male, 3 females; stn. C5, 2 males, 4 females, 2 ovigerous females. COBERPES 1 stn. A9, 1 male, 1 female, 1 ovigerous female; stn. B9, 5 males, 4 females, 1 ovigerous female; stn. B14, 1 female, 1 ovigerous female; stn. Ω2, 11 males, 8 females, 9 ovigerous females; stn. Ω15, 2 males, 3 females; stn. E4, 1 female. COBERPES 2011 stn. E1, 2 ovigerous females; E7, 4 males. COBERPES 3 stn. α5, 1female; stn. B10, 2 males, 1 female. COBERPES 4 stn. A1b, females 1; A3, 1 male; stn. B15b, 7 males, 5 females; stn. B26b, 7 males, 7 females; stn. B27, 1 male; stn.C33b, 7 females; stn. C34, 4 males, 2 females.

###### Additional material examined.

SIGSBEE 9 stn. A4, 8 males, 5 females, 1 ovigerous female, stn. A6, 15 males, 11 females, 5 ovigerous females, stn. A7, 11 males, 1 female, 1 ovigerous female, stn. A9, 11 males, 14 females, 4 ovigerous females. SIGSBEE 10 stn. C, 1 male, stn. D, 4 females, stn. E, 2 females, stn. F, 1 male, 1 ovigerous female.

###### Remarks.

This species presented a wide distribution in the southern Gulf of Mexico, in Yucatán, off Celestún; Tamaulipas, San Fernando River; Veracruz, Tamiahua Lagoon and Pánuco River; in Tabasco off Grijalva-Usumacinta Rivers; and Quintana Roo, off Holbox Island; from 110.5 to 1140.0 m depth. *Agononida longipes* was the most common and abundant species of squat lobsters throughout all cruises with 446 individuals. The overall sex ratio was 0.94 M: 1.0 F. The maximum abundance was observed in summer (77.8%; from 309.0 to 1140.0 m) and in the SSW sector (53.4%; 231.6–913.0 m). Ovigerous females (n = 58), exhibited larger mean CL x = 15.328 ± 2.148 (min. 10.7, max. 20.8 mm) than males (n = 216), CL x = 14.253 ± 6.583 (min. 2.0, max. 34.0 mm) and females (n = 172), CL x = 14.048 ± 3.025 (min. 7.61, max. 22.5 mm).

**Figure 3.**
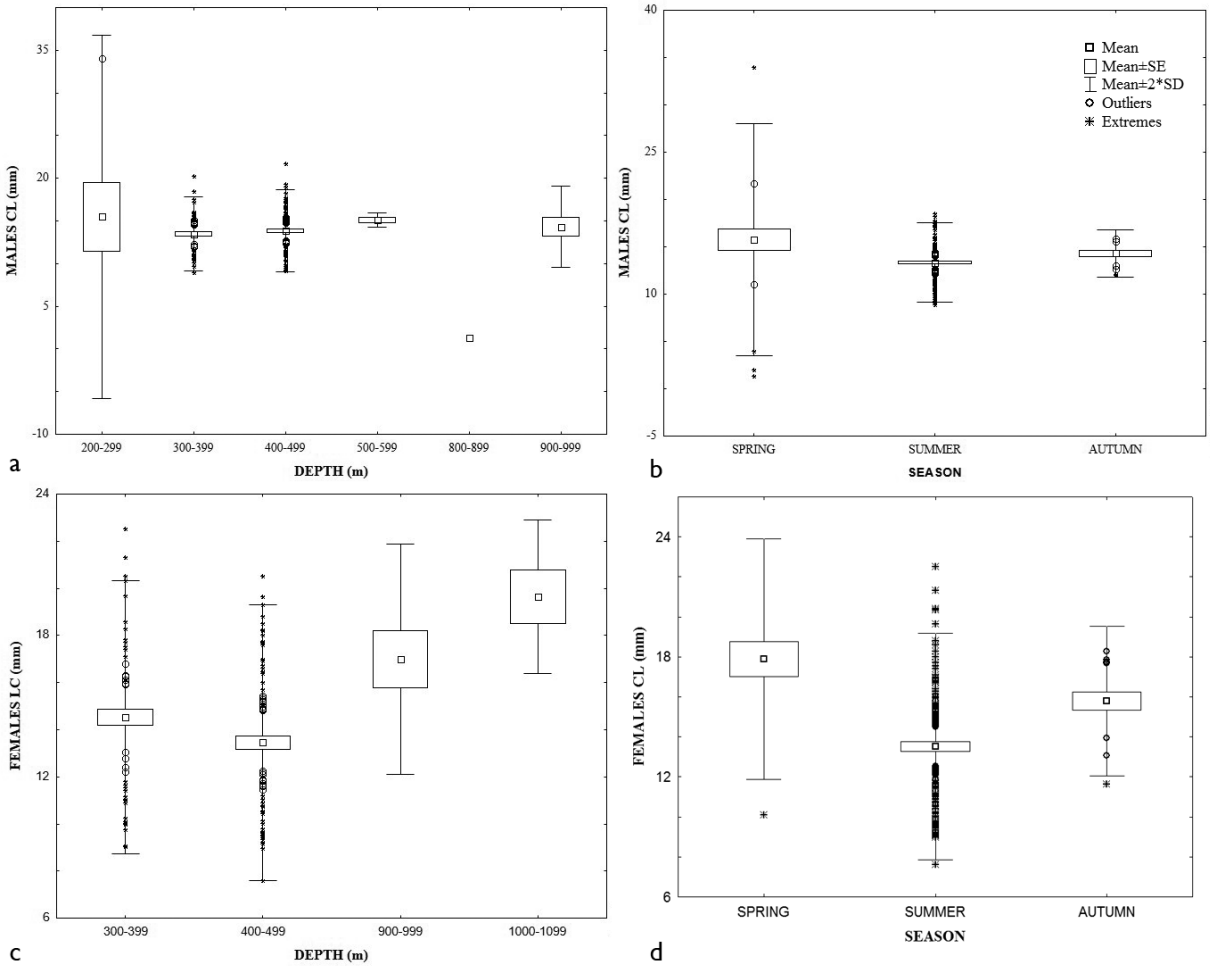
Size distribution of *Agononida longipes*, males: **a** depth interval season; females **c** depth interval **d** seasons; ovigerous females **e** season.

The ANOVA test showed that the mean CL in males, females, and ovigerous females were statistically different among seasons: F (2;207) = 8.48; p = 0.00; F (3;168) = 5.83; p = 0.00; F (2;55) = 6.94; p = 0.00, respectively. The largest sizes were present in spring, whereas the smallest were in summer (Figs 3b, d, e; Tukey Test). However, the mean CL among the depth strata were statistically different only in males and females: F (5;204) = 4.49; p = 0.00; F (3;168) = 5.83; p = 0.00, respectively. The largest males were collected in shallower depths (Fig. 3a), while the largest females were in deeper ones (Fig. 3c). The seasonal sex ratio approached the expected proportion (1 M: 1 F), except in autumn (0.68 M: 1 F), but it was not statistically different (χ^2^ = 1.524, p = 0.2170). Ovigerous females were present in spring, summer, and autumn, and the largest number was collected in summer between 269.0–913.0 m depth. The percentage of bopyrid infestation in each sex was relatively uniform (males = 5.8 and females 4.1). The CL size of parasitized individuals ranged from 1.9 to 15.2 mm in males and 11.1 to 15.4 mm in females.

##### Munida constricta

Taxon classificationAnimaliaDecapodaMunididae

A. Milne-Edwards, 1880

###### Material examined.

BATO stn. 53, 3 males. BIOREPES 1: stn 6, 1 male; stn 42, 3 males, 1 female; stn. 47, 4 males, 2 females, 1 ovigerous female; stn 50, 2 males, 1 female; stn 54, 2 males, 3 females, 1 ovigerous female; stn 55, 8 males, 5 females. BIOREPES 2 stn 4, 1 male; stn. 11, 1 male; stn 12, 1 male, stn. 25, 13 males, 2 females; stn. 31, 3 males; stn. 37, 4 males. COBERPES 3 stn. B2, 2 males.

###### Remarks.

**Figure 4.**
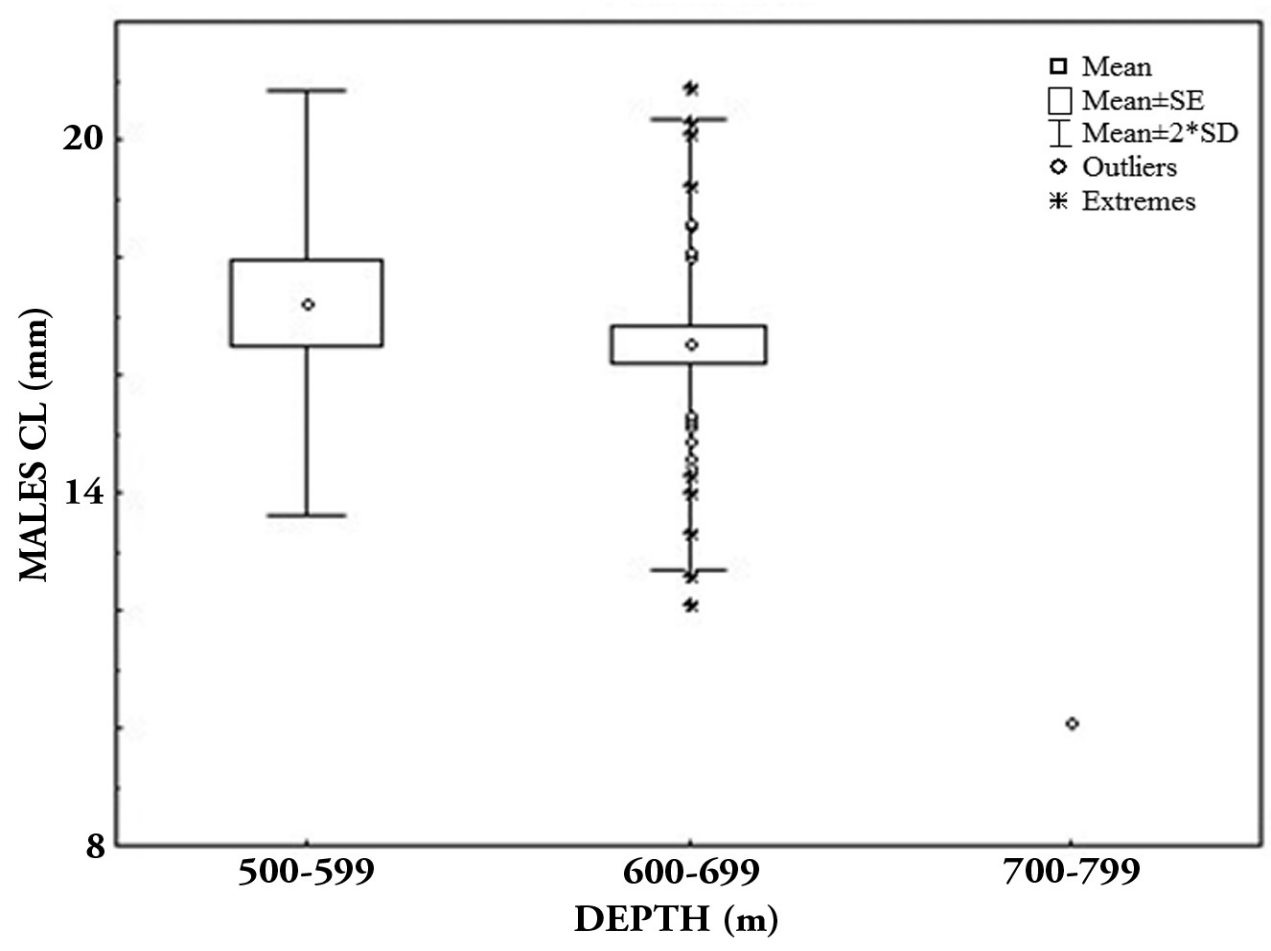
Size distribution of *Munida constricta*, males and depth interval.

We collected 62 individuals in the sectors SSW, SSE, and ESE; from 305.3 to 814.0 m depth. In Tabasco, this species was found off Carmen y Machona Lagoon; Yucatán at N of Alacranes Reef, and N of Celestún; and in Campeche, off Términos Lagoon. The highest abundance was found in summer (93.5%) at a 321.4–717.8 m depth range. *Munida constricta* was mainly reported in sector SSE (79.1%, 536.0–717.0 m). The overall sex ratio was 3.2 M: 1 F, χ^2^ = 16.516, p < 0.0001. Females exhibited larger mean CL x = 14.1 ± 3.02 (min.7.6- max. 22.5 mm) than males CL x = 13.7 ± 2.516 (min. 2.0, max. 21.7 mm). Only the CL size of males presented statistically significant differences (ANOVA: F (2; 45) = 6.08; p = 0.00); the smallest sizes were found at shallower depths, whereas larger ones were observed at deeper depths (Fig. 4). The sex ratio in summer (3.8 M: 1 F) was significantly different from the expected proportion (χ^2^ = 19.931, 1 degree of freedom, two-tailed, P = 0.0001). The two ovigerous females (CL = 13.6–17.6 mm) were collected in autumn. We found only one female (CL = 14.43 mm) infested with bopyrid. The material collected represents the first records in the sectors SSW, SSE, and ESE.

##### Munida evermanni

Taxon classificationAnimaliaDecapodaMunididae

Benedict, 1901

###### Material examined.

BATO stn. 32, 1 male. BIOREPES 1 stn.5, 12 males, 6 females, 2 ovigerous females, stn 6, 19 males, 9 females, 9 ovigerous females 9, stn. 8, 2 males, 1 female, 1 ovigerous female, stn, 20 10 males, 3 females, 8 ovigerous females, stn 22, 2 males. BIOREPES 2 stn. 14, 12 males, stn. 16, 8 males, 1 female, 8 ovigerous females, stn. 18, 2 males, 1 female, 15 ovigerous females, stn. 21, 2 males, 1 ovigerous female, stn. 32, 1 ovigerous female. COBERPES 2011 stn. E1, 30 males, 29 ovigerous females. COBERPES 3 stn. B9, 2 males.

###### Remarks.

**Figure 5.**
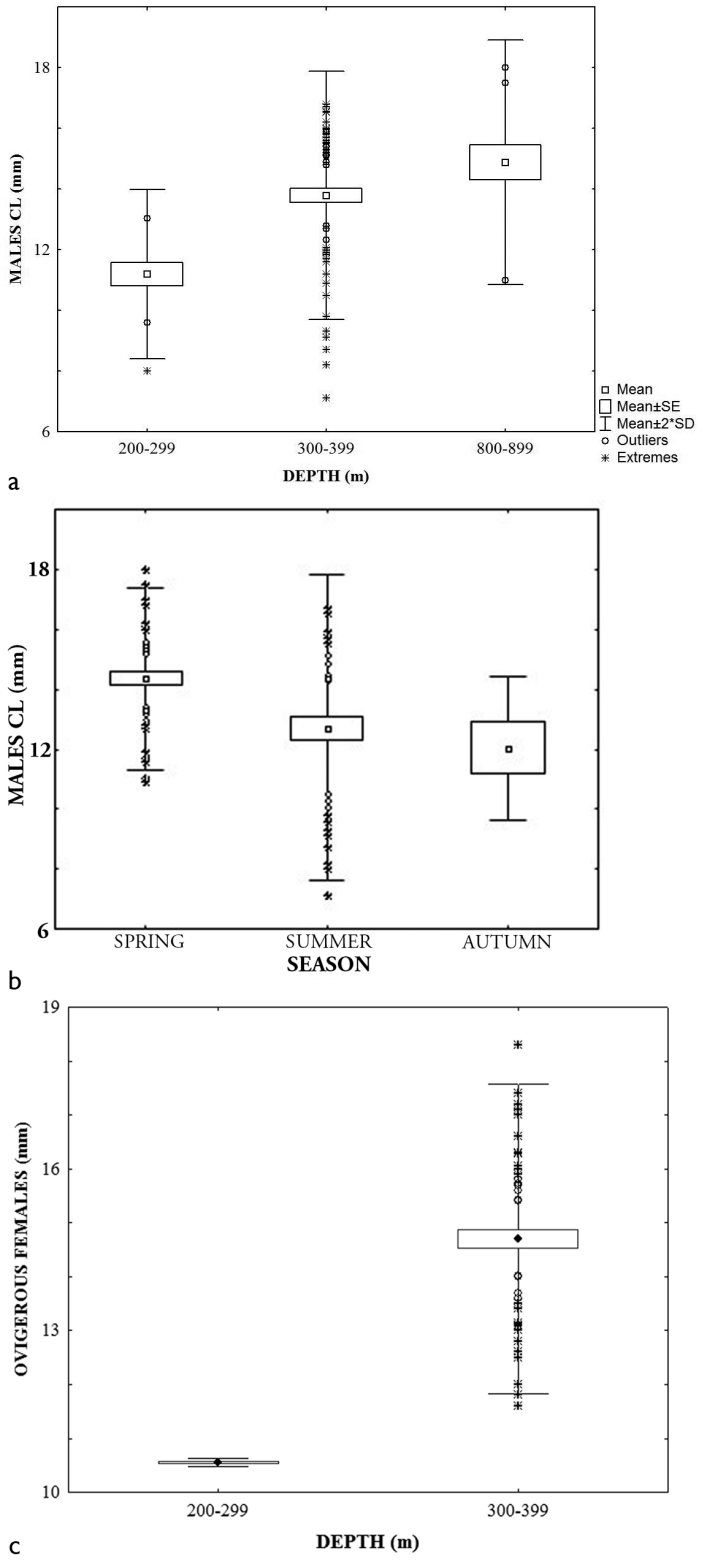
Size distribution of *Munida evermanni*, males: **a** depth interval **b** season; ovigerous females **c** season.

This species was the third one in abundance with 197 individuals. They were collected in sectors SSW, WSW and SSE, from Yucatán: N of Celestún, N Alacranes Reef, Puerto Progreso to Campeche, off Términos Lagoon. The maximum abundance was observed in summer (68.5%; 257.4–863.0 m) mainly in the SSE sector (55.8%; 305.3–346.0 m). The overall sex ratio (1.07 M: 1 F) was not statistical significantly different. The mean CL size of ovigerous females was larger x = 14.6 ± 1.569 (min. 10.5, max. 18.3 mm) than males x = 13.6 ± 2.189 (min. 7.1, max. 18.0 mm) and females x = 13.5 ± 2.747 (min. 9.7, max. 19.4 mm). The smallest CL male sizes were observed in shallower depths, while the largest ones were found in deeper areas (F = 12.52; p = 0.00) (Fig. 5a). The smallest sizes were mainly reported in spring and the largest in autumn (F = 8.69; p = 0.00) (Fig. 5b). The ovigerous females were collected in spring and summer, and we found a significant difference in CL by depth (F = 16.46; p = 0.00) (Fig. 5c). The sex ratio in spring and summer presented minimum differences that were not statistically significant. The material collected represents the first records in the sectors SSW, WSW, SSE. In addition, we increase the deeper bathymetric range to 863 m.

##### Munida forceps

Taxon classificationAnimaliaDecapodaMunididae

A. Milne-Edwards, 1880

###### Material examined.

BATO stn. 11, 1 female, stn. 32, 1 male. BIOREPES 1 stn 15, 3 males, 3 females, 2 ovigerous females. BIOREPES 3 stn. A1, 2 ovigerous females, stn A 24, 2 males.

###### Additional material examined.

MOPEED 1 stn. J1, 1 male. MOPEED 2 stn. J2, 2 males, 1 ovigerous female, stn. W1, 1 female. MOPEED 4 stn. W1, 1 male, stn. W2, 1 male. SGM 7 stn. GO8, 2 ovigerous females. SGM 8 stn. 6.7, 1 male. SGM 10 stn. 91.26, 1 male. SIGSBEE 9 stn. A9, 1 ovigerous female. SIGSBEE 10 stn F, 1 female.

###### Remarks.

*Munida forceps* was collected in the Campeche Bank, Campeche; Tuxpan, Veracruz; and off Alacranes Reef, Yucatán from 55 to 442.5 m. The highest abundance was observed in the SSW sector (77.8%, 55 to 269 m), mainly in summer (70.4%); the overall sex ratio was 0.93 M: 1 F. Females were larger x = 15.476 ± 1.862 (13.7–18.1 mm) than males x = 14.356 ± 3.471 (10.2–20.2 mm) and ovigerous females x = 13.776 ± 4.524 (7.6–20.5 mm). The ovigerous females were present in summer and autumn.

##### Munida iris

Taxon classificationAnimaliaDecapodaMunididae

A. Milne-Edwards, 1880

###### Material examined.

BATO stn. 29, 2 males; stn. 33, 1 ovigerous female; stn. 41, 3 males, 2 females; stn. 49, 1 male; stn. 50, 2 males, 1 female; stn. 54, 1 male, 2 ovigerous females; stn. 59, 1 male, 1 ovigerous female. BIOREPES 1 stn, 12, 2 males, 2 ovigerous females; stn. 27, 33 males, 26 females, 42 ovigerous females; stn.30, 2 males, 1 female; stn 34, 37 males, 1 female, 1 ovigerous female. BIOREPES 2 stn. 2, 1 female; stn 10, 1 ovigerous female. BIOREPES 3 stn. A1, 1 female; stn. A2, 1 ovigerous female; stn. A24, 1 male; stn. B2, 1 male; stn. B6, 1 male; stn. C5, 2 males, 2 females, 1 ovigerous female. COBERPES stn. B9, 2 males; stn. Ω2, 1 male, 1 female. COBERPES 3 stn. B2, 3 males; stn. B9, 3 males; stn. B10, 2 males, stn B 15B, 1 ovigerous female. COBERPES 2011 stn. B9, 3 males, 1 ovigerous female, stn. E1, 1 male; stn E4, 2 males, 2 ovigerous females; stn. E7, 8 males, 1 ovigerous female. COBERPES 4 stn. B15B, 1 male, 1 ovigerous female; stn C34B, 1 male.

###### Additional material examined.

SIGSBEE 9 stn. A9, 1 male. MOPEED 2 stn. W1, 1 female.

###### Remarks.

**Figure 6.**
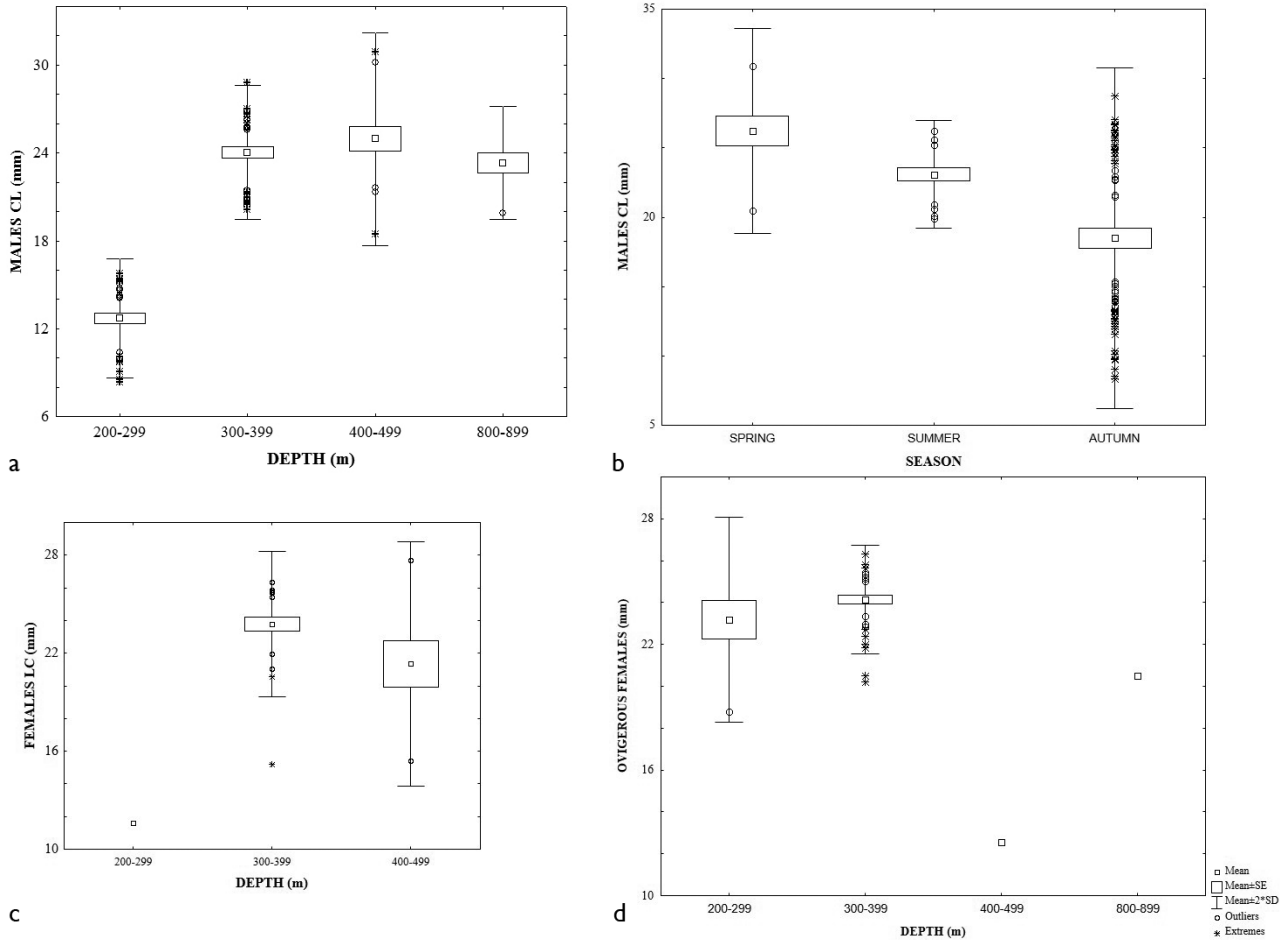
Size distribution of *Munida iris*, males: **a** depth interval **b** season; females: **c** depth interval; ovigerous females **d** depth interval.

This species was the second in abundance with 215 individuals collected off San Fernando River, Tamaulipas; Tuxpan, Veracruz; Campeche Bank, Campeche; off Alacranes and Arenas Reef, Yucatán; sectors: WNW, WSW, SSW, ESE, SSE; 244.6–1040.0 m. The overall sex ratio was 1.26 M: 1 F. The greatest abundance was found in summer (69.8%; 244.6–913.0 m) mainly in the ESE sector (54.9%; 249.9–452.0 m). The ovigerous CL mean was larger (x = 23.7 ± 2.74, min. 12.6, max. 26.3 mm) than females (x = 22.9 ± 3.304 min. 11.6, max. 27.7 mm) and males (x = 20.2 ± 6.041 min. 8.3, max. 30.9 mm). The ANOVA analysis of CL showed significant differences through depth strata in all sexes: males F (3; 97) = 162.55, p = 0.00, females F (2; 33) = 12.60, and ovigerous females F _depth_ (3, 48) = 21.80, p = 0.00. Male and ovigerous female small sizes were found at shallow depth interval, whereas females were at deeper depth (Figs 6a, c, d). Only males presented significant size difference among seasons F (2;98) = 13.59; p = 0.00. Highest sizes were reported in autumn, whereas small ones were in summer (Fig. 6b). The differences of sex ratio in spring (1.07 M: 1 F) and summer (2.3 M: 1 F) were not statistically different compared to autumn (3.25 M: 1 F; χ^2^ = 4.765, with 1 degree of freedom, two-tailed P = 0.0290). Ovigerous females were collected in spring, summer, and autumn; the greatest percentage (20.9%) was reported in summer in a 245–412 m depth range. Two males were infested with rhizocephalans (CL 21.3–24–9 mm), also one male (CL = 23.8 mm) and one female (CL = 30.3 mm) were infested with bopyrid.

##### Munida irrasa

Taxon classificationAnimaliaDecapodaMunididae

A. Milne-Edwards, 1880

###### Material examined.

BIOREPES1 stn. 34, 11 ovigerous females; stn. 35, 1 male.

###### Remarks.

*Munida irrasa* occurred off Alacranes Reef, Yucatán; SSW, 443.7–829.0 m depth. The ovigerous females size ranged from 9.6 to 12.2 mm (summer at 443.7m), and the only male’s CL size reported was 13.1 mm (summer at 829.0 m).

##### Munida microphthalma

Taxon classificationAnimaliaDecapodaMunididae

A. Milne-Edwards, 1880

###### Material examined.

COBERPES stn. A6, 1 male.

###### Remarks.

We collected only one male (CL = 12.5 mm) in front of Carmen y Machona Lagoon, Tabasco; 1144 m, sector WSW.

##### Munida miles

Taxon classificationAnimaliaDecapodaMunididae

A. Milne-Edwards, 1880

###### Material examined.

BATO stn. 10, 1 male, 1 ovigerous female, stn. 29, 1 male, stn. 54, 1 male, 2 ovigerous females, stn. 59, 1 male. BIOREPES 1 stn 12, 2 males, 2 females, 3 ovigerous females, stn 13, 1 male, 1 ovigerous female, stn 18, 1 female 1, stn 28 1 female, stn 31, 1 male. BIOREPES 2 stn 10, 1 male. COBERPES 2011 stn. B4, 1 male, 1 ovigerous female. COBERPES 3 stn B2, 1 female, stn. B10, 3 males, 1 female, 1 ovigerous female.

###### Remarks.

*Munida miles* was collected off Alacranes Reef, Yucatán; Campeche Bank, Campeche; San Fernando River, Tamaulipas; at 245.6–590.0 m. Although, it was most abundant in sector SSW (89.7%; 327.5–590.0 m), mainly in summer (51.7%; 394.5–455.8 m). Sex ratio throughout seasons was similar and close to the expected proportion 1:1. The mean CL in ovigerous females was 18.7 mm, 18.1 mm for males and 14.1 mm for females. The ovigerous females (31.09% of the total collected) occurred in spring, summer, and autumn, but the maximum number was found in summer. Only one male was infested with a rhizocephalan parasite (CL = 18.9 mm). The material collected represents the first record in the sector SSW.

##### Munida valida

Taxon classificationAnimaliaDecapodaMunididae

Smith, 1883

###### Material examined.

BATO stn. 10, 1 ovigerous female; stn. 35, males 1, females 1, ovigerous females 1; stn. 48, 1 male, 1 female; stn. 49, 2 males, 1 female; stn. 53, males 2, females 3. BIOREPES 1 stn. 42, 3 males, 3 females; stn 47, 5 males, 5 females; stn 48, 2 males, 1 female; stn 50, 3 males; stn 54, 1 male, 3 females; stn 55, 9 females. BIOREPES 2 stn. 10, 1 female; stn.11, 3 males, 1 ovigerous female; stn. 25, 2 males, females 2; stn. 37, 1 male, 1 female. BIOREPES 3 stn. A12, 6 males, 2 ovigerous females; stn. A13, 2 males; stn. A24, 2 males; stn. B1, 1 ovigerous female; stn. B5, 1 female, 1 ovigerous female; stn. C7, 2 males. COBERPES stn. A3, 2 males; stn. A12B, males 2; stn. B2, 1 male; stn. B12, 1 female; stn. B13, 1 male, 1 female; stn. B15, 1 male, 1 female; stn. Ω1, 2 males; stn. Ω6, 2 males; stn. Ω7, 2 females; stn. Ω8, 1 male; stn. Ω14, 1 male; stn. Ω15, 1 female. COBERPES 2011 stn. B2, 1 male; stn. C2, 1 female; stn. D11, 9 males, 8 females; 7 ovigerous females. COBERPES 3 stn. B11, 2 males; stn. α5, 2 males; stn. α6, 1 male; stn. α7, 1 female; stn. α10, 2 males, 1 female, 1 ovigerous female. COBERPES 4 stn A5, 1 female; stn C35B, 1 female; B12B, 1 female; stn B 13, 2 males, 1 female; stn B 14, 2 males, 1 female; stn 30 B, 2 males.

###### Remarks.

This species was frequent in the catches (i.e., 140 individuals), and was practically present in all sectors of the southern Gulf of Mexico: N Celestún, Holbox, Progreso, N Alacranes, Yucatán; Carmen y Machona Lagoons, Tupilco Lagoon, San Pedro and San Pablo Rivers, Tabasco; Coatzacoalcos, Veracruz; 359–1048 m. However, the greatest number was collected in the SSE (n = 44, 536.0–700.0 m) and SSW (n = 42, 317.5–780.0 m) sectors, particularly off rivers and lagoons. The major percentage of organisms (52.9%) was reported in summer (359.0 to 770.0 m), whereas less one was recorded in autumn (10.7%). Ovigerous females mean CL (x = 29.8 ± 7.770, min. 15.4, max. 41.7) was larger than males mean CL, (x = 27.3 ± 7.629 min. 9.7, max. 40.2), and females mean CL (x = 26.2 ± 6.471 min. 11.0, max. 44.0). The smallest size of males and females were found mainly at deeper strata. The ANOVA results were not significant for males CL: [F _depth_ (5; 65) = 1.22, p = 0.31; F _season_ (2, 68) = 0.07, p = 0.93], and females CL: [F _depth_ (3; 50) = 1.05, p = 0.37; F _season_ (2, 51) = 1.31, p = 0.27]. The sex ratio in autumn (2.4 M: 1 F) showed significant differences (χ^2^ = 4.48, p = 0.03).

Ovigerous females (n = 15) were present in all seasons and almost in all sectors (except WNW) in a depth interval of 510 to 642 m. Four males (CL = 24.5–40.2 mm) and four females (CL = 21.1–44.4 mm) were infected with rhizocephalan. Also one male (CL = 24.3 mm) and one female (CL = 32.5 mm) were infected with bopyrid.

#### Family Munidopsidae

We collected 285 organisms belonging to two genera and 18 species of Munidopsidae. Only two species had sample size large enough to perform statistical analyses.

##### Galacantha spinosa

Taxon classificationAnimaliaDecapodaMunidopsidae

A. Milne-Edwards, 1880

###### Material examined.

BIOREPES 1 stn. 28, 1 male, stn. 48, 1 ovigerous female. BIOREPES 2 stn. 5, 1 ovigerous female, stn. 7, 1 ovigerous female, stn 14, 1 ovigerous female, stn. 25, 3 males, stn. 36, 1 male, stn. 37, 1 male. BIOREPES 3 stn. A14, 1 male, 1 female, stn. A15, 3 males, 1 female. COBERPES stn. A6, 1 male, stn. α5, 1 male, stn. α 7, 2 males, 1 ovigerous female, stn. α 8, 1 male, 1 ovigerous female. COBERPES 2011 stn. B2, 1 male, stn. C4, 1 male, 1 ovigerous female, stn. C5, 1 ovigerous female, stn D1, 2 males, 1 female, 1 ovigerous female. COBERPES 3 stn. B17, 1 ovigerous female, stn α7, 2 males, 1 ovigerous female, stn. α 11, 2 males, 1 female, 1 ovigerous female. COBERPES 4 stn. B11, 1 ovigerous female.

###### Remarks.

*Galacantha spinosa* was collected off Laguna Madre, Tamaulipas (WNW); Tuxpan, Veracruz (WSW), Términos Lagoon, and San Pedro and San Pablo rivers, Campeche (SSW); 640–1144 m. This species was most abundant in the SSW sector and in autumn (42.5% and 37. 5%, respectively). Overall sex ratio favored males 1.4: 1 F, but this difference was not statistically significant. Females reached larger sizes CL x - = 34.3 ± 4.798 (min. 27.0, max. 43.0 mm) than males x = 30.5 ± 7.111. (min. 14.8, max. 40.0). The ovigerous females (CL = 23.6 to 35.4 mm) occurred in spring, summer, and autumn at a 735–1016 m depth range. One female (CL = 20.5 mm) was infested by bopyrid. These are the first records in sectors WSW and WNW; also, we increase the deeper bathymetric limit to 1144 m.

##### Munidopsis abbreviata

Taxon classificationAnimaliaDecapodaMunidopsidae

(A. Milne-Edwards, 1880)

###### Material examined.

BIOREPES 2 stn. 27, 1 female, stn. 28, 1 ovigerous female.

###### Remarks.

The only two females found (CL = 13.1, and ovigerous = 19.3 mm) were collected in summer. This constitutes the first record in the Gulf of Mexico, off Alacranes Reef, Yucatán; 828.9–965.3 m (sectors SSW and SSE). These records also increase the shallow bathymetric range to 829 m.

##### Munidopsis alaminos

Taxon classificationAnimaliaDecapodaMunidopsidae

Pequegnat & Pequegnat, 1970

###### Material examined.

BIOREPES 1 stn. 48, 1 male, 1 ovigerous female, stn. 25, 1 male. COBERPES stn. E2, 1 female. COBERPES 2011 stn. C4, 1 ovigerous female. COBERPES 3 stn. α7, 1 female. COBERPES 4 stn. B14, 1 male, stn. A5, 1 female, 1 ovigerous female.

###### Remarks.

The specimens collected in this study were found at San Fernando River, Tamaulipas; Grijalva-Usumacinta Rivers, Tabasco; off Tupilco Lagoon, Tabasco; off Holbox Island, Quintana Roo; in a 513–735 m depth range. The male CL size ranged from 9.8 to 13.9 mm, and the females size ranged from 7.0 to 11.3 mm. Ovigerous females (10.1 to 10.3 mm) were present in spring and summer at 700–735 m. This material constitutes the first record in the SWS (513.0- 640.0 m) and SSE (700.0 m) sectors. One male (CL = 10.3 mm) was infested with bopyrid.

##### Munidopsis armata

Taxon classificationAnimaliaDecapodaMunidopsidae

(A. Milne-Edwards, 1880)

###### Material examined.

BIOREPES 2 stn. 23, 1 male. COBERPES stn. B8, 1 ovigerous female. COBERPES 2011 stn. B1, 2 males, 2 ovigerous females, stn. B9, 4 males, 2 females, 3 ovigerous females.

###### Remarks.

We collected 15 individuals in front of Grijalva-Usumacinta Rivers, Tabasco; Campeche Bank, Campeche; at 560.0–1040.0 m. Females CL range was 10.8–10.9 mm, whereas the CL for males varied between 5.0–12.3 mm. The ovigerous females (CL = 8.9 to 11.9 mm) were collected in summer at 976.0–1040.0 m. It is the first time that this species is recorded in the SSE and SSW sectors. It was previously reported in sector ESE; Caribbean and South America (Felder et al. 2009).

##### Munidopsis bradleyi

Taxon classificationAnimaliaDecapodaMunidopsidae

Pequegnat & Pequegnat, 1971

###### Material examined.

COBERPES stn. Ω14, 1 male.

###### Remarks.

This species was found off Tupilco, Lagoon, Tabasco, 573 m depth in the SSW sector. The only individual collected (CL = 25.1 mm) constitutes the first record for the Gulf of Mexico, it was previously recorded in the Caribbean Sea (Baba et al. 2008).

##### Munidopsis erinacea

Taxon classificationAnimaliaDecapodaMunidopsidae

(A. Milne-Edwards, 1880)

###### Material examined.

BATO stn. 42, 1 male. BIOREPES stn. 47, 11 males, 3 females, 7 ovigerous females 7, stn. 55, 1 ovigerous female. BIOREPES 2 stn. 4, 1 male 1, stn. 11, 2 males, stn. 24, 1 female, stn. 32, 1 male. BIOREPES 3 stn. B6, 2 males. COBERPES stn. A11, 1 female, stn. Ω7, 1 female, stn. A12b, 1 male, 1 ovigerous female, stn B2, 1 ovigerous female, stn. B12, 1 male, stn Ω7, 1 female, stn. Ω14, 1 female. COBERPES 2011 stn. B2, 1 ovigerous female, stn. B4, 2 males, 1 female, 1 ovigerous female, stn. C2, 4 males, 4 ovigerous females, stn. C3, 2 males, 1 ovigerous female, stn. C4, 1 male, 1 female, stn. D1b, 1 female, stn. D6, 1 female, 1 ovigerous female, stn. D9, 11 males. COBERPES 3 stn. α 10, 1 male, stn. α 11, 1 ovigerous female, stn. B17, 1 male.

###### Remarks.

It was collected off Soto la Marina River, Tamaulipas; San Pedro and San Pablo Rivers, Tupilco Lagoon, Tabasco; Términos Lagoon, Campeche; N Alacranes, Yucatán; (ESE, SSE, SSW, WSW); 406.0–820 m. *Munidopsis erinacea* was the sixth species in terms of abundance (n = 72). The highest abundance was observed in the sector ESE during the spring season in a depth range of 700–799 m. The ANOVA analysis was made only for males (n = 37), but did not show significant differences by depth [F (3;32) = 1.1295; p = 0.3518] or season [F (2;33) = 3.0006; p = 0.0635]. The mean size of males was x = 14.0 ± 3.954 (6.0–21.4) at 406–780 m depth range. The mean size of females was x = 10.4 ± 3.346 (6.4–18.0) at 530–820 m depth. The mean size of ovigerous females was x = 12.7 ± 2.431 (8.3–17.1) at 530–820 m depth.

The ovigerous females were observed in all seasons; however, they were more numerous during spring in the ESE sector. Sex ratio was similar in all seasons (1 M: 1 F). Two individuals were infested by rhizocephalans (CL male = 15.9 mm, female = 17.0), and one female (CL = 13.1 mm) was infested by bopyrid.

##### Munidopsis latifrons

Taxon classificationAnimaliaDecapodaMunidopsidae

(A. Milne-Edwards, 1880)

###### Material examined.

COBERPES 2011 stn. D9, 1 female.

###### Remarks.

Only one specimen was observed in southern Gulf of Mexico: Holbox Island, Yucatán (ESE), at 769 m depth. The female was collected in spring (CL = 5.3 mm).

##### Munidopsis longimanus

Taxon classificationAnimaliaDecapodaMunidopsidae

(A. Milne-Edwards, 1880)

###### Material examined.

COBERPES 3 stn. α 10, 1 male.

###### Remarks.

We collected only one male during autumn (CL = 10.7 mm), off Grijalva-Usumacinta Rivers, Tabasco (sector SSW) at 780.0 m depth.

##### Munidopsis polita

Taxon classificationAnimaliaDecapodaMunidopsidae

(A. Milne-Edwards, 1880)

###### Material examined.

COBERPES 4 stn. C33B, 1 male.

###### Remarks.

We collected only one individual during summer (CL = 6.4 mm); additionally, this record is the first one for the WSW sector (Off San Fernando River, Tamaulipas); 352.0 m depth.

##### Munidopsis ramahtaylorae

Taxon classificationAnimaliaDecapodaMunidopsidae

Pequegnat and Pequegnat, 1971

###### Material examined.

COBERPES stn. A6, 1 female.

###### Remarks.

The only one female was caught in summer (CL = 10.7 mm) and constitutes the first record in the SSW sector (Grijalva-Usumacinta Rivers, Tabasco at 495 m).

##### Munidopsis riveroi

Taxon classificationAnimaliaDecapodaMunidopsidae

Chace, 1939

###### Material examined.

BIOREPES 3 stn. A10, 1 male.

###### Additional material examined.

SIGSBEE 9 stn. A4, 1 ovigerous female.

###### Remarks.

We collected two individuals off Laguna Madre, Tamaulipas; and off Tamiahua Lagoon, Veracruz (WSW) from 344.5 to 351.0 m depth. We captured one male (CL = 3.6 mm) in autumn and one ovigerous female in summer (CL = 7.4 mm). Both data represent the first record of the species in the entire Gulf of Mexico.

##### Munidopsis robusta

Taxon classificationAnimaliaDecapodaMunidopsidae

(A. Milne-Edwards, 1880)

###### Material examined.

BIOREPES 3 stn. A2, 4 males, 8 ovigerous females; stn A11, 1 female, 1 ovigerous female; stn. A12, 1 male; stn. B1, 2 ovigerous females; stn. B3, 1 male, 1 ovigerous female; stn. B4, 1 male; stn. B6, 10 males, 2 females, 7 ovigerous females. COBERPES stn. A4, 1 female, 1 ovigerous female; stn. Ω 15, 2 males, 1 ovigerous female. COBERPES 2011 stn. D7, 1 male. COBERPES 3 stn. α 7, 1 male. COBERPES 4 stn A3, 1 ovigerous female; stn A4, 2 males, 2 ovigerous females; stn A5, 11 males, 10 females; stn B15, 1 male; stn B15B, 9 males, 1 female, 10 ovigerous females; stn C35, 1 female.

###### Remarks.

**Figure 7.**
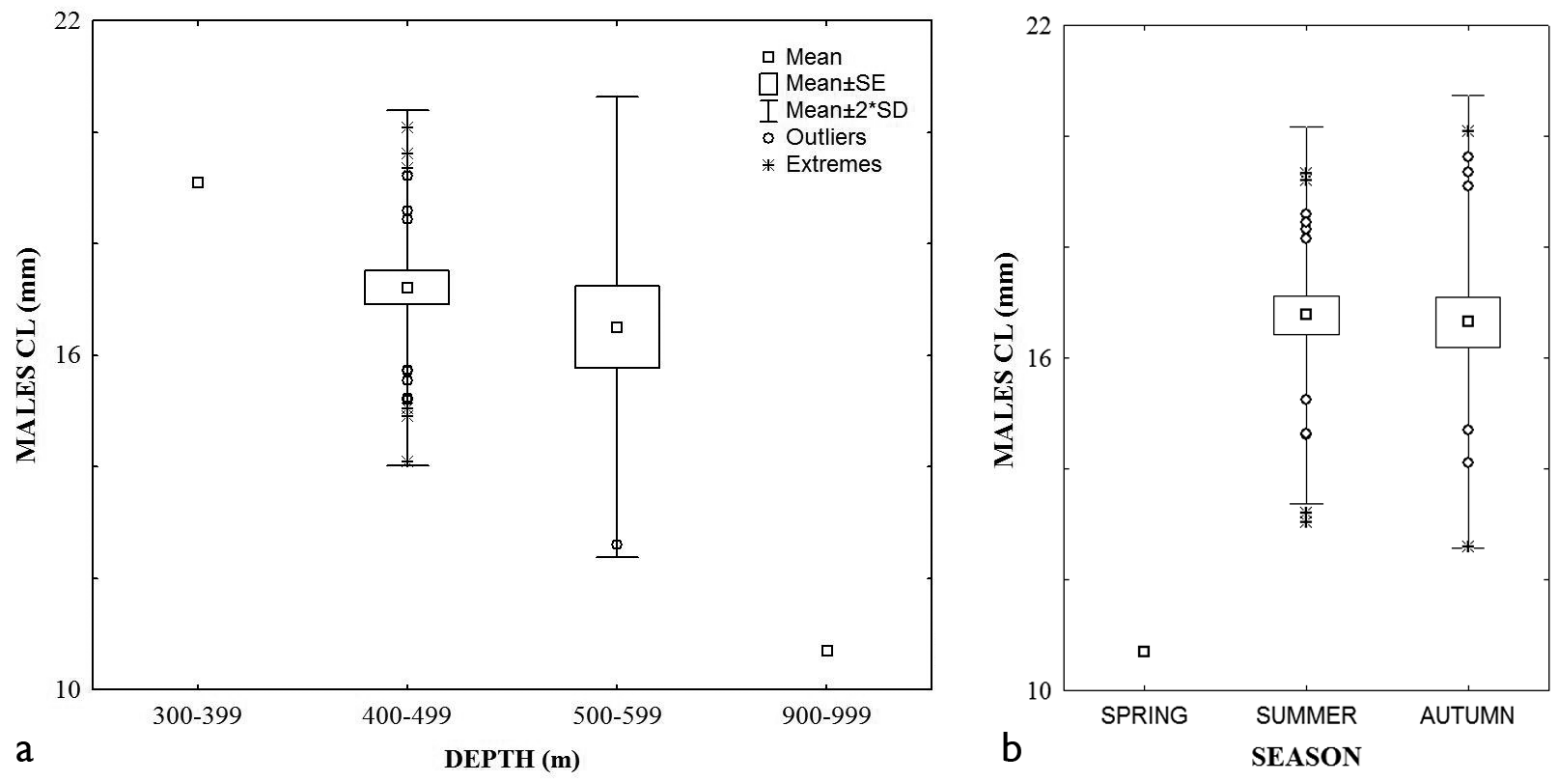
Size distribution of *Munida robusta*, males: **a** depth interval **b** season.

*Munidopsis robusta* was commonly captured in the Gulf of Mexico from the N Carolina–Florida Straits; Gulf of Mexico (all sectors); Caribbean Sea-Colombia (Ortega-Echeverría 2014); 110–4708 m (Felder et al. 2009). In the southern part, we collected this species, off N and S of Laguna Madre (sectors WNW, WSW); Soto La Marina and San Fernando River (WSW); Mecoacán Lagoon and Tonalá River (SSW); and Cabo Catoche (ESE), from 347.0 to 953.0 m depth. This species was the most abundant of the Munidopsidae family (n = 97 individuals). Many of them were collected in the WNW sector (53.9%; 401.3–546.0 m) during autumn (52.6%; 347.0 to 577.0 m). The CL mean size of ovigerous females was larger [x = 18.4 ± 2.129 (14.0–22.6)] than males [x = 16.9 ± 1.983 (10.7–20.1)]. The males’ size was small at shallow depths and also during autumn (F_(depth)_ = 5.434, p = 0.0037, and F_(season)_ = 8,956, p = 0.0007) (Fig. 7a, b).

The overall sex ratio difference was not statistically different (χ^2^ = 0.051, p = 0.8208); as it was observed among seasons. The ovigerous females occurred in spring, summer, and autumn in a depth interval of 347.0–546.0 m. Maximum number was reported in autumn. One male (CL = 13.4 mm) was infested by bopyrid.

##### Munidopsis serratifrons

Taxon classificationAnimaliaDecapodaMunidopsidae

(A. Milne-Edwards, 1880)

###### Material examined.

BIOREPES 1 stn 28, 1 male. COBERPES 2011 stn D9, 1 male.

###### Remarks.

This species has been reported in the Gulf of Mexico in Florida (NNW, ESE); Caribbean, Dominica, and Bermuda; 543–1967 m (Baba et al. 2008; Felder et al. 2009). Only two organisms of *Munidopsis serratifrons* were collected off Ría Lagartos, and Cabo Catoche, Yucatán (SSW, ESE) at a 614.0–769.0 m depth range. This species occurred in summer and spring. Male CL = 10.2, female CL = 9.8 mm.

##### Munidopsis serricornis

Taxon classificationAnimaliaDecapodaMunidopsidae

(Lovén, 1852)

###### Material examined.

BIOREPES 1 stn. 48, 2 males, 2 females, 1 ovigerous female. COBERPES 2011 stn B9, 3 males, 1 female, 1 ovigerous female, stn D9, 2 males, 4 females.

###### Remarks.

*Munidopsis serricornis* has been captured off Georgia; Gulf of Mexico (sector SE); West and East Atlantic; Indo West Pacific; 10?–2165 m (Felder et al. 2009). In the present study it was only collected in front of Ría Lagartos, Yucatán (ESE). Most of the individuals were collected in spring at 769.0–1040.0 m depth. The CL size ranges were: males (6.0–12.7mm), females (6.0–10.6 mm), and ovigerous females (7.8–10.7 mm). The two ovigerous females occurred in spring and summer (1040.0 and 700.0 m depth, respectively).

##### Munidopsis shulerae

Taxon classificationAnimaliaDecapodaMunidopsidae

Vázquez- Bader, Gracia & Lemaitre, 2014

###### Material examined.

BATO stn. 15, 1 male. BIOREPES 1 stn. 55, 1 male. BIOREPES 2 stn. 25, 1 female, stn. 31, 1 female.

###### Remarks.

This species has been reported in the northern and southern part of the Gulf of Mexico: Straits of Florida; off coast of Campeche, and the western Caribbean from 320 to 787 m (Vázquez-Bader et al. 2014). In the present study *Munidopsis schulerae* was collected off N Celestún, and N Alacranes, Yucatán (sector ESE) from 503 to 640 m depth. The two males analyzed had a CL size range of 6.8–10.5 and females 7.9–10.4 mm.

##### Munidopsis sigsbei

Taxon classificationAnimaliaDecapodaMunidopsidae

(A. Milne-Edwards, 1880)

###### Material examined.

BIOREPES 2 stn. 7, 1 male, stn. 8, 3 females, 1 ovigerous female, stn. 28, 1 female. COBERPES stn. B9, 1 male, 1 ovigerous female, stn. Ω10, 1 female. COBERPES 2011 stn. B9, 2 males, 1 female, 2 ovigerous females. COBERPES 3 stn. α1, 1 male. COBERPES 4 stn. A 6, 1 male, stn. B 9, 1 male, 2 ovigerous females.

###### Remarks.

*Munidopsis sigsbei* presented a wide distribution: from the Gulf of Mexico to Brazil in depths of 500 to 2000 m (Kilgoure and Shriley 2014). Specimens of this study were collected in Yucatán: N Alacranes and N Celestún; Veracruz: off Coatzacoalcos River; and Tabasco: in front of San Pedro and San Pablo Rivers (sectors SSE and SSW). *Munidopsis sigsbei* was the third in terms of abundance (n = 19). Males had a CL range of 10.3–20.6 mm, females showed a 7.4–15.6 mm CL range, and ovigerous females a 10.8–15.3 mm CL range. Ovigerous females occurred in spring and summer.

##### Munidopsis simplex

Taxon classificationAnimaliaDecapodaMunidopsidae

(A. Milne-Edwards, 1880)

###### Material examined.

COBERPES 2011 stn. C5, 1 male.

###### Remarks.

*Munidopsis simplex*, has been previously reported in all sectors of Gulf of Mexico; Caribbean Sea; Eastern Atlantic; from 116–3971 m (Felder et al. 2009); 458–1830 m (Baba et al. 2008); and 1000–3968 m (Kilgoure and Shirley 2014). Specimen in this analysis was found off Holbox Island, Yucatán (ESE) at 806 m depth. The only male (CL = 17.9 mm) was collected in spring.

##### Munidopsis spinoculata

Taxon classificationAnimaliaDecapodaMunidopsidae

(A. Milne-Edwards, 1880)

###### Material examined.

BIOREPES 1 stn. 3, 1 female. BIOREPES 3 stn. D1, 1 female. COBERPES 1 stn. Ω7, 1 male.

###### Remarks.

The records for this species include Florida Straits; Gulf of Mexico (sectors: NNE, SW, and ESE); Caribbean Sea; 597– 1738 m (Mayo 1974). In the southern Gulf of Mexico, we collected this species off Alacranes Reef; Tamiahua Lagoon, Tamaulipas; Grijalva-Usumacinta Rivers, Tabasco (sectors WSW, SSW) from 400.4 to 750.0 m depth. The only male had 10.2 mm CL; whereas females were 10.7–11.2 mm CL, respectively.

## Discussion

A total of 1513 squat lobsters were collected: Chirostylidae (n = 95), Galatheidae (n = 2), Munidopsidae (n = 285), and Munididae (n = 1131) belonging to 6 species of Chirostyloidea and 27 of Galatheoidea. The low abundance of Chirostylidae may due to its specific habitat requirements (living associated with corals and other anthozoans, further than 1000 m) that are difficult to access and sample (Pequegnat 1983; Baba 2005; Le Guilloux et al. 2010; Schnabel et al. 2011b). However, *Uroptychus nitidus* was the most abundant and frequent species of the family. This was previously pointed out by other studies in the Gulf of Mexico (e.g., Kilgoure and Shirley 2014). However, the scarce abundance of Galatheidae could be an artifact of depth range sampled, as *Galathea rostrata*, mainly distributes at an upper depth range (< 200 m).

A comprehensive analysis of the abundance showed that four species of the Munididae family contributed to 64.7% of the total organisms collected. These were in order of abundance: *Agononida longipes*, *Munida iris*, *Munida evermanni*, and *Munida valida*. Some authors, like Creasy et al. (2000) and Kilgoure and Shirley (2014), also mentioned *Agononida longipes* as the most common and abundant species in the northern part of the Gulf of Mexico. Other species like *Munidopsis robusta*, *Munidopsis erinacea*, *Munida constricta*, and *Uroptychus nitidus*, although represent a low percentage (17.7%), were a common and frequent component in the capture. The highest abundance of many species occurred in front of river mouths or lagoons, particularly in the Campeche Bank and off Alacranes Reef areas.

Galatheoid and chirostyloid species occurs in a wide bathymetrical range, 0 to 5400 m, which indicates they have overlapping depth distribution ranges (Schnabel et al. 2011b). In this study we found differences in bathymetrical distribution among the families. The Munididae were mainly found in the 300–399 m depth interval, whereas the highest abundances of Munidopsidae and Chirostylidae were present in 400–499 m and 1000–1099 m, respectively. We extend the bathymetric range for nine species (shallower and deeper). We also extend the spatial range in three species of Chirostylidae (*Gastroptychus salvadori*, *Uroptychus capillatus*, and *Uroptychus spiniger*). We reported for the first time three species of Munidopsidae (*Munidopsis bradleyi*, and *Munidopsis riveroi*) in the Gulf of Mexico (Table 1).

**Table 1.** Chirostyloidea and Galatheoidea species distribution in Gulf of Mexico, Caribbean, and Brazil. CAR = Caribbean; BRA = Brazil; NE = Northeast (nne = north northeast, ene = east northeast); NW = Northwest (nnw = north northwest; wnw = west northwest); SE = Southeast (sse = south southeast; ese = east southeast); SW = Southwest (wsw = west south west; ssw = south southwest). **^1^** = New record for Gulf of Mexico; ∆ = New record for sector; + = Extension of bathymetric range.

	GULF OF MEXICO								CAR	BRA	DEPTH (m)
SPECIES	NE		NW		SW		SE				
	nne	ene	nnw	wnw	wsw	ssw	sse	ese			
*Eumunida picta*	x		x	x				x	x		200–600
*Gastroptychus affinis*		x						x	x		78–635
*Gastroptychus meridionalis*										x	358–800
*Gastroptychus salvadori* **^1^**						∆		∆	x		650–874 +
*Gastroptychus spinifer*	x		x		∆			x	x		212–2412
*Uroptychus aguayoi*									x		528
*Uroptychus armatus*									x		298
*Uroptychus brevis*								x	x		457–1107
*Uroptychus capillatus* **^1^**							∆	∆	x		306–1040 +
*Uroptychus fornicatus*									x		555.6
*Uroptychus intermedius*									x		298
*Uroptychus jamaicensis*								x	x		677–1249
*Uroptychus minutus*				x					x	x	46–137
*Uroptychus nitidus*	x	x	x	x	x	x	x	x		x	161–1342
*Uroptychus princeps*									x		514
*Uroptychus rugosus*								x	x		174–549
*Uroptychus spiniger* **^1^**				∆				∆	x		708–1040 +
*Uroptychus spinosus*								x	x		265–421
*Uroptychus uncifer*			x	x		∆		x	x	x	155–1144 +
*Galathea rostrata*	x	x	x	x		∆	x	x			18–159
*Agononida longipes*	x	x	x	x	x	x	x	x	x	x	40–1140 +
*Agononida schroederi*								x	x		274–531
*Agononida caribensis*						x			x		11.0–38.0
*Munida affinis*	x				x	x		x	x		42–914
*Munida angulata*	x	x	x	x	x	x	x	x	x	x	24–260
*Munida atlantica*										x	58–166
*Munida beanii*	x								x		39–78
*Munida benedicti*									x		174–430
*Munida chacei*				x					x		393–446
*Munida coltroi*										x	240–260
*Munida constricta*	x	x				∆	∆	∆	x	x	200–549 +
*Munida elfina*									x		670
*Munida evermanni*			x		∆	∆	∆	x	x		232–863 +
*Munida flinti*	x	x	x	x	x	x	x	x	x	x	11–641
*Munida forceps*	x	x	x	x	x	x	x	x	x	x	40–950
*Munida heblingi*										x	83
*Munida iris*	x	x	x	x	x	x	x	x	x	x	40–1303
*Munida irrasa*	x	x	x	x			x	x	x	x	38–914
*Munida media*						x		x	x		500–536
*Munida microphthalma*	x	x	x	x	x	x	x	x	x	x	195–2412
*Munida miles*		x				∆		x	x	x	68–659
*Munida nuda*		x							x		68–630
*Munida petronioi*										x	75
*Munida pusilla*	x	x	x	x	x	x	x	x	x	x	7–200
*Munida robusta*									x	x	298
*Munida santipauli*								x		x	18–2360
*Munida sculpta*								x	x		179–284
*Munida serrata*									x		329–421
*Munidopsis simplex*	x	x	x	x	x	x	x	x	x		16–440
*Munida spinifrons*	x							x		x	13–260
*Munida stimpsoni*	x							x	x		172–897
*Munida striata*								x	x		274–503
*Munida subcaeca*									x		842–1700
*Munida valida*	x	x	x	x	x	x	x	x	x	x	279–2297
*Munida victoria*										x	960
*Galathea rostrata*	x								x	x	1600–3800
*Galacantha spinosa*			x			x		x	x		183–1144 +
*Munidopsis agassizii*									x		300–1642
*Munidopsis abbreviata*				x		∆	∆	x	x		860–1342 +
*Munidopsis abdominalis*		x							x		350–720
*Munidopsis alaminos*	x			x	∆		∆				428–842
*Munidopsis aries*			x	x							71–5320
*Munidopsis armata*						∆	∆	x	x		275–1446
*Munidopsis barbarae*		x							x	x	185–200
*Munidopsis bermudezi*		x		x					x		2434–5180
*Munidopsis bradleyi* **^1^**						∆			x		485–600
*Munidopsis brevimanus*								x	x		366–906
*Munidopsis colombiana*									x		4151–4153
*Munidopsis crassa*		x						x	x		1026–5315
*Munidopsis cubensis*								x	x		759–1144
*Munidopsis curvisostra*									x		146–2430
*Munidopsis erinacea*	x	x	x	x	x	x	x	x	x	x	238–1574
*Munidopsis espinis*								x	x		779–897
*Munidopsis expansa*								x	x		457–1107
*Munidopsis geyeri*	x	x			x				x		2600–4151
*Munidopsis gilli*								x	x		1638–2139
*Munidopsis glabra*			x								510–622
*Munidopsis granulens*									x		347–353
*Munidopsis gulfensis*				x	x				x		1097–1400
*Munidopsis kucki*											227
*Munidopsis latifrons*								x	x		677–1107
*Munidopsis livida*	x								x		2070–3496
*Munidopsis longimanus*	x		x	x	x	x	x	x	x		292–1281
*Munidopsis nitida*	x				x	x			x	x	592–3968
*Munidopsis penescabra*				x							543–807
*Munidopsis platirostris*								x	x		91–842
*Munidopsis polita*			x	x	∆	x		x	x	x	129–1170
*Munidopsis ramahtaylorae*	x	x				∆			x		200–668
*Munidopsis reynoldsi*									x		4086–4277
*Munidopsis riveroi* **^1^**					∆				x	x	260–3822
*Munidopsis robusta*	x	x	x	x	x	x	x	x	x		79–4708
*Munidopsis serratifrons*			x					x	x		325–1966
*Munidopsis serricornis*			x				x	x	x		200–2165
*Munidopsis sharrei*									x		298–454
*Munidopsis shulerae*	x	x	x	x		x		x			320–787
*Munidopsis sigsbei*	x	x	x	x	x	x	x	x	x	x	500–2000
*Munidopsis similis*		x									1475–2438
*Munidopsis simplex*	x	x	x	x	x	x	x	x	x		116–3971
*Munidopsis spinifer*						x		x	x		203–880
*Munidopsis spinoculata*	x			x	x	x		x	x		778–1738
*Munidopsis squamosa*									x		212–500
*Munidopsis subspiniculata*						x			x		457–823
*Munidopsis transtridens*								x			1162–1475
*Munidopsis tridens*								x	x		380–600

Only eight species had enough number to stand statistical analyses, the rest of the species presented, each one, an abundance minor than 30 individuals. However, the ANOVA was only statistically significant for *Uroptychus nitidus*, *Agononida longipes*, *Munida constricta*, *Munida evermanni*, *Munida iris*, and *Munidopsis robusta*. Males of *Uroptychus nitidus* and *Munida evermanni* were larger in spring, whereas *Munida iris and Munidopsis robusta*, were larger in autumn. In *Agononida longipes*, females presented the major size in the deeper strata, whereas in *Munida iris* they were found in the shallower ones. Kilgour and Shirley (2014), mentioned that ovigerous females were significantly larger than females in: *Uroptychus nitidus*, *Galacantha spinosa*, *Munidopsis abbreviata*, *Munidopsis alaminos*, *Munidopsis erinacea*, *Munidopsis robusta*, *Munidopsis sigsbei*, and *Munidopsis simplex*. In many of the species studied here, the largest ovigerous females occurred in the SSW and WSW sectors, which are subjected to the influence of rivers and lagoons.

The overall sex ratio difference was only statistical significant for two species: *Munida constricta* and *Uroptychus capillatus*; while Bursey (1978); and Wenner (1982), reported significant differences in *Agononida longipes*, *Munida valida*, *Munida bairdii*, and *Munida iris*.

In terms of seasonal occurrence, 58% of all species were present in summer, 30% in spring, and 12% in autumn. A high percentage of males and females (47.6%) occurred in summer, whereas ovigerous females (13.2%) occurred mainly in spring. However, ovigerous females were also collected during summer and spring, suggesting that families like Galatheoidea and Chirostyloidea do not have a marked seasonal reproduction, as pointed out by Kilgoure and Shirley (2014).

The incidence of parasitism in our study was low. Only eight species, that represents 4.7% of the total individuals of Galatheoidea were parasitized. Almost 88% of these individuals were infested by rhizocephalan barnacles, and 11% by bopyrid isopod, particularly in summer. *Agononida longipes* was the most heavily parasitized by rhizocephalan barnacles (3% of infestation incidence), all individuals were captured in only one station in the Alacranes Reef area. Pequegnat (1983) reported for this species one organism infected by bopyrid and one by rhizocephalan barnacle in the Northern Gulf of Mexico. In addition, she found other species like *Munida iris* and *Munida rostrata* infected with bopyirid and rhizocephalan, whereas Wenner and Windsor (1979) mentioned a 2.2–5.0% infestation incidence by bopyrid isopods in *Munida iris*, but Williams and Brown (1972) reported 10% for the same species.

Global diversity studies of squat lobsters (Schnabel et al. 2011b) revealed that the Western Atlantic including the Caribbean, Brazil, and Gulf of Mexico, reported 10.2–12.1% of Chirostyloidea and 10.9–15.4 of Galatheoidea (Campos et al. 2005; Fierro et al. 2008; Felder et al. 2009; Kilgoure and Shirley 2014; de Melo-Filho 2006, de Melo-Filho and de Melo 2001 a, 2001 b; Navas et al. 2003; Ortega et al. 2014) (Table 1). Nevertheless, the global diversity records of these two groups are incomplete, 19–20% of Chirostylidae and 37–38% of Galatheidae species remain undescribed, particularly in tropical offshore areas (Appeltans et al. 2012). We reported 51.4% of Munidopsidae species, 33.3% of Munididae species, and 80% of Chirostylidae species known for the Gulf of Mexico. It is worth mentioning, that 13.8% of the species recorded in this study were only reported in the Caribbean Sea.

At this moment, a total of 71 species has been reported for the entire Gulf of Mexico. Munididae were dominant in the SSW sector whereas Munidopsidae and Chirostylidae were more abundant in the ESE sector. The SE sector is the most important one with 54 species, NE with 35, NW 31, and SW with 26. In terms of biodiversity of the southern Gulf of Mexico we found in our study that the subsector ESE presented the higher number of species with restricted distribution and 18.3% of the total collected were only reported here, 2.8% in the SSW, and 1.4 in WSW. In the SE sector, 80% of the chirostylids and 25.6% of the munidopsids showed higher range restrictions, compared with 14.3% of muninids. According to Felder et al. (2009) and Baba et al. (2008), the NNE, NNW, and WNW had 7.0% of species with restricted distribution, but ENE had 5.6%. The high percentage of chirostylids restricted occurrence is perhaps related to its limited dispersal potential compared to the other galatheoids (Schnabel et al. 2011b); and also with the fact, that these species could have more specific habitat, as they are predominantly associated with corals and other anthozoans (Baba 2005).

## Supplementary Material

XML Treatment for Gastroptychus salvadori

XML Treatment for Gastroptychus spinifer

XML Treatment for Uroptychus capillatus

XML Treatment for Uroptychus nitidus

XML Treatment for Uroptychus spiniger

XML Treatment for Uroptychus uncifer

XML Treatment for Galathea rostrata

XML Treatment for Agononida longipes

XML Treatment for Munida constricta

XML Treatment for Munida evermanni

XML Treatment for Munida forceps

XML Treatment for Munida iris

XML Treatment for Munida irrasa

XML Treatment for Munida microphthalma

XML Treatment for Munida miles

XML Treatment for Munida valida

XML Treatment for Galacantha spinosa

XML Treatment for Munidopsis abbreviata

XML Treatment for Munidopsis alaminos

XML Treatment for Munidopsis armata

XML Treatment for Munidopsis bradleyi

XML Treatment for Munidopsis erinacea

XML Treatment for Munidopsis latifrons

XML Treatment for Munidopsis longimanus

XML Treatment for Munidopsis polita

XML Treatment for Munidopsis ramahtaylorae

XML Treatment for Munidopsis riveroi

XML Treatment for Munidopsis robusta

XML Treatment for Munidopsis serratifrons

XML Treatment for Munidopsis serricornis

XML Treatment for Munidopsis shulerae

XML Treatment for Munidopsis sigsbei

XML Treatment for Munidopsis simplex

XML Treatment for Munidopsis spinoculata
